# Association of the *COQ2* V393A Variant with Parkinson's Disease: A Case-Control Study and Meta-Analysis

**DOI:** 10.1371/journal.pone.0130970

**Published:** 2015-06-22

**Authors:** Xinglong Yang, Jing Xi, Quanzhen Zhao, Hua Jia, Ran An, Zhuolin Liu, Yanming Xu

**Affiliations:** 1 Department of Neurology, West China Hospital, Sichuan University, 37 Guo Xue Xiang, Chengdu, Sichuan Province, 610041, P.R. China; 2 Department of Neurology, First Affiliated Hospital, Sun Yat-sen University, Guangzhou, Guangdong Province, 510080, P.R. China; National Institutes of Health, UNITED STATES

## Abstract

Both Parkinson’s disease (PD) and multiple system atrophy (MSA) are neurodegenerative diseases of uncertain etiology, but they show similarities in their pathology and clinical course. The fact that the gene encoding α-synuclein is associated with both diseases also suggests that they share some genetic determinants. Recent studies in Japan associating MSA with a variant in the *COQ2* gene led us to question whether variants in the *COQ2* gene are associated with PD in Han Chinese in a case-control study. A total of 564 patients with PD were genotyped using the ligase detection rection, together with 484 gender- and age-matched healthy subjects. The M128V and R387X variants of *COQ2* were not detected in patients or controls; instead, we detected only the heterozygous V393A variant (CT genotype). The frequency of the CT genotype encoding the V393A mutation was significantly higher in patients PD (4.08%) than in controls (1.86%), corresponding to an odds ratio of 2.24 (95%CI 1.03 to 4.90, p = 0.037). The frequency of the C allele of the V393A variant was significantly higher in patients with PD than in controls (OR 2.22, 95%CI 1.02 to 4.82, p = 0.039), and this was also observed in a meta-analysis of studies from mainland China, Taiwan and Japan. Subgroup analysis of our data showed that the V393A variant was significantly associated with early-onset PD (OR 3.71, 95%CI 1.51 to 9.15, p = 0.002) but not with late-onset disease (OR 1.65, 95%CI 0.69 to 3.95, p = 0.260). Gender was not significantly associated with either genotype or minor allele frequencies. In conclusion, our findings show for the first time that the V393A variant in the *COQ2* gene increases risk of PD among the population of east Asia. These results, combined with research on Japanese, lend genetic support to the hypothesis that oxidative stress underlies pathogenesis of both PD and MSA.

## Introduction

Multiple system atrophy (MSA) and Parkinson’s disease (PD) are neurodegenerative diseases that share several common features in their pathogenesis and their clinical manifestations. In both diseases, the characteristic pathological hallmark is aggregation of α-synuclein, encoded by the *SNCA* gene; however, the etiology of both disorders is unclear [1]. PD is recognized as a gene-associated disease, and pedigree and genome-wide association studies have established links with mutations in *SNCA*, *DJ-1*, *GBA*, *LRRK2* and other genes [2]. In fact, a mouse model of PD carrying the *SNCA* transgene has been established [3]. MSA, originally thought to be a sporadic disease with no familial transmission, has been associated with both autosomal recessive and autosomal dominant inheritance patterns in pedigrees [3]. Since these initial studies, MSA has been linked to several genes, including *interleukin (IL) 21A*, *IL-1B*, *IL-8*, *tumor necrosis factor*, *α-1-antichymotrypsin*, *SLC1A4*, *SQSTM1* and *EIF4EBP1* [3–5].

Evidence that MSA and PD may share genetic determinants came with the announcement of an association of MSA with the *SNCA* variants rs3822086 and rs3775444 [6]. Several members of a British family who have PD and who express a G51D variant of α-synuclein also show neuropathological features characteristic of MSA [7]. These findings suggest that α-synuclein dysfunction may be common to PD and MSA. They also raise the question of whether MSA and PD share other genetic determinants apart from *SNCA* polymorphism.

A study of familial MSA in Japan recently identified a homozygous mutation in the *COQ2* gene (M128V-V393A/M128V-V393A). They also identified heterozygous *COQ2* mutations encoding single substitutions (V393A) or double substitutions (R387X/V393A). The *COQ2* gene product is an enzyme that participates in the synthesis of coenzyme Q10, and the polymorphism that leads to the V393A enzyme variant leads to lower enzyme expression and therefore lower production of coenzyme Q10 [8]. As a result, individuals expressing this variant produce more free radicals and are less able to clear them from tissues, increasing oxidative stress that ultimately leads to apoptosis. Since oxidative stress is already known to play an important role in the pathogenesis of both MSA and PD, we wondered whether these *COQ2* variants may be associated with risk of PD. Indirect evidence for this has come from studies showing that serum from patients with PD has significantly lower coenzyme Q10 concentrations than serum from healthy individuals, and that coenzyme Q10 supplementation shows promise for treating PD [9].

In order to explore possible overlap in genetic determinants of PD and MSA and in the corresponding pathophysiological mechanisms, we conducted a case-control study in Han Chinese. We examined whether the homozygous mutation M128V-V393A/M128V-V393A, the compound heterozygous mutation R387X/V393A and the single heterozygous mutation V393A are associated with PD.

## Subjects and Methods

### 2.1 Subjects

Han Chinese patients with sporadic PD (302 men, 262 women) were consecutively recruited from the movement disorder center of West China Hospital, Sichuan University. Their mean age was 62.64±10.83 years; patients were divided into a group with late-onset PD (LOPD; n = 397) if the age at onset was >50 years (mean age at onset, 63.75±6.63), and a group with early-onset PD (EOPD; n = 167), in which the mean age at onset was 44.98±4.66. PD was diagnosed in all patients independently by two movement disorder specialists according to criteria for idiopathic PD from the UK PD Society Brain Bank [10]. Only sporadic patients were included; patients with at least one relative with PD were excluded.

To provide a control group to compare with the entire PD group as well as with the EOPD and LOPD subgroups separately, we recruited 484 healthy Han Chinese (288 men, 196 women; mean age, 61.94±11.53) who were unrelated to the PD patients (A large proportion of the controls were the spouses of the patients, meanwhile, all of them were unrelated individuals without a past or family history of neurodegenerative disease) and who were age- and gender-matched with them. his study protocol was approved by the Ethics Committee of West China Hospital, Sichuan University. All study participants provided written informed consent.

### 2.2 *COQ2* genotyping

Genomic DNA was obtained from peripheral leukocytes by classic phenol-chloroform extraction. Genotyping of various *COQ2* polymorphisms was then performed by the Shanghai BioWing Applied Biotechnology Company using the ligase detection reaction (Table 1) [11]. Briefly, target DNA sequences were amplified using multiplex PCR, then 1 μl of proteinase K (20 mg/ml) was added to each amplification reaction, which was incubated at 70°C for 10 min and quenched at 94°C for 15 min. The ligation reaction for each study subject was carried out in a final volume of 20 μl containing 20 mM Tris-HCl (pH 7.6), 25 mM potassium acetate, 10 mM magnesium acetate, 10 mM DTT, 1 mM NAD, 0.1% Triton X-100, 10 μl of multiplex PCR product, 1 pmol of each discriminating oligo, 1 pmol of each common probe and 0.5 μl of 40 U/μl Taq DNA ligase (New England Biolabs, USA). Finally, the ligase detection reaction was performed using 40 cycles of 94°C for 30 s and 63°C for 4 min. Fluorescent ligation products were separated and analyzed on an ABI Sequencer 377.

**Table 1:** Summary of *COQ2* variants genotyped by ligase detection reaction (LDR) in Han Chinese patients with Parkinson's disease.

Variant	Base change	Primer	LDR product (bp)
V393A	C/T	GCTGTTTTCTCCTCCGTGTT(forward) TTCCACAAATTCCCAAGGAC (reverse)	206
M128V	A/G	CACGGTGGTGACTTGCAG(forward) GACTCGGAGGCTGCTACTTG (reverse)	195
R387X	A/G	GCTGTTTTCTCCTCCGTGTT(forward) TTCCACAAATTCCCAAGGAC (reverse)	206

### 2.3. Statistical analysis

Age was reported as mean ± SD, and gender, allele and genotype frequencies were reported as percentages. Allele and genotype frequencies were determined by direct counting of *COQ2* alleles. Concordance of genotype distribution with Hardy-Weinberg equilibrium (HWE) was verified, and genotype frequencies were compared using the chi-squared test. Associations of gender with particular alleles and genotypes were also assessed by chi-squared test, whereas age differences among the three groups were assessed using the t test. Two-tailed P < 0.05 was considered significant. Since only one variant(V393A) was involved in our research, there is no need to perform the multiple test or multiple correction. SPSS 17.0 (IBM, Chicago, USA) was used to perform all statistical analysis, including chi-squared and t tests.

## Results

The PD patients and controls showed similar distributions of age (χ^2^ = 3.167, p = 0.160) and gender (T = 0.709, p = 0.492) (Table 2). We detected only the heterozygous polymorphism encoding the V393A substitution; we did not detect the M128V or R387X variant in either group, thus only the V393A variant was detected as a common variant in the present research. The distribution of genotypes at this polymorphism was in accordance with HWE for the PD group (χ^2^ = 0.244, p = 0.621) and controls (χ^2^ = 0.043, p = 0.836). The frequency of the CT genotype for the V393A variant was significantly higher in patients (4.08%) than in controls (1.86%) (Table 2). Effect size estimation showed a significantly higher frequency for the CT genotype in the PD group relative to controls (OR 2.24, 95%CI 1.03 to 4.90, p = 0.037). The C allele of this variant occurred more frequently in the PD group than in controls (OR 2.22, 95%CI 1.02 to 4.82; χ^2^ = 4.26, p = 0.039) (Table 3).

**Table 2:** Demographic characteristics of Han Chinese patients with Parkinson’s disease (PD) and age-matched, healthy Han Chinese controls.

Characteristic	PD group(n = 564)	Control group(n = 484)	Comparison
Age, yr	62.64±10.83	61.94±11.53	T = 0.709, p = 0.492
Gender, n			
Male	302	288	χ^2^ = 3.167, p = 0.160
Female	262	196	

**Table 3:** Genotype and minor allele frequencies for *COQ2* polymorphism in subgroups of Han Chinese patients with Parkinson's disease.

Group		Genotype		OR (95%CI); *P*	Allele		OR (95%CI); *P*
		CT	TT		C	T	
*Patients with PD*							
All	*564*	23 (4.08)	541 (95.92)	2.24 (1.03 to 4.90); 0.037*	23 (2.04)	1105 (97.96)	2.22 (1.02 to 4.82); 0.039*
Males	302	10 (3.31)	292 (96.68)	1.94 (0.65 to 5.74); 0.224**	10 (1.66)	594 (98.34)	1.92(0.65 to 5.66); 0.227**
Females	262	13 (4.96)	249 (95.4)	2.51 (0.80–7.81); 0.102**	13 (2.48)	511 (97.52)	2.47 (0.80 to 7.63);0.105**
EOPD	167	11 (6.59)	156 (93.41)	3.71 (1.51 to 9.15); 0.002*	11 (3.29)	323 (96.71)	3.63 (1.49 to 8.84); 0.002*
LOPD	397	12 (3.02)	385 (96.98)	1.65 (0.69 to 3.95); 0.260*	12 (1.51)	782 (98.49)	1.64 (0.69 to 3.90); 0.263*
*Controls*							
All	484	9 (1.86)	475 (96.19)		9 (0.93)	959 (99.07)	
Males	288	5 (1.74)	283 (98.26)		5 (0.86)	571 (99.13)	
Females	196	4 (2.04)	192 (97.96)		4 (1.02)	388 (98.97)	

CI, confidence interval; EOPD, early-onset Parkinson's disease; LOPD, late-onset Parkinson's disease; OR, odds ratio; PD, Parkinson’s disease.

* Compared with entire control group.

** Compared with subgroups of male or female controls.

To test whether other factors may be influencing the observed relationship between *COQ2* polymorphism and risk of PD, we compared genotype and minor allele frequencies in PD subgroups based on gender or age at onset. Gender was not significantly associated with genotype or minor allele frequencies (Table 3). Comparing the EOPD subgroup and controls showed significant differences in frequencies of genotypes (p = 0.002) and minor allele (p = 0.002) (Table 3). In contrast, no significant differences were observed when the LOPD subgroup was compared with controls (Table 3).

## Discussion

This case-control study in Han Chinese patients with PD shows for the first time that the V393A variant is associated with PD. Given that this variant is associated with reduced production of coenzyme Q10 [8] and therefore with increased oxidative stress, our findings provide evidence that oxidative stress may play a key role in the onset and progression of PD.

The *COQ2* gene encodes a biosynthetic enzyme involved in the production of coenzyme Q10, which is located in the inner mitochondrial membrane, where it serves as an electron acceptor for complex I (NADH-ubiquinone oxidoreductase) and complex II (succinate-ubiquinone oxidoreductase) of the mitochondrial electron transport chain. It also serves as an antioxidant [12]. Coenzyme Q10 deficiency impairs mitochondrial function and leads to increased redox cycling of the limited coenzyme Q10 pool, generating more semiquinone and thereby increasing the production of reactive oxygen species [13]. Oral administration of coenzyme Q10 to animals provides dose-dependent neuroprotection against striatal lesions induced by the complex II inhibitor malonate [14]. The V393A variant of *COQ2* leads to lower production of coenzyme Q10 [8,13], resulting in an increase in oxidative stress and compromising the body's natural defenses against reactive oxygen species. Our findings are consistent with previous suggestions that oxidative stress is associated with onset and/or progression of α-synucleinopathies including MSA and PD, based on the observation that superoxide induces α-synuclein aggregation in vitro [15].

**Fig 1:**
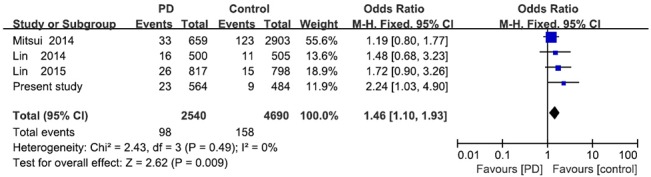
Meta-analysis of the association between the *COQ2* V393A variant and PD in different Asian populations. The *Events* column reports the number of patients with the CT genotype; the *Total* column, the total number of patients with CC and CT genotypes.CI, confidence interval; OR, odds ratio; PD, Parkinson’s disease.

This is the first study to confirm an association between PD and the *COQ2* V393A variant. One study in Japan [8] and two from Taiwan [16,17] failed to detect this association, although they did find a trend towards higher frequency of the C allele in the PD group than in the control group: 2.5% vs. 1.6–2.2% in the Japanese study, and 1.6% vs. 0.94–1.1% in the Taiwanese work. When we meta-analyzed the data from these three studies with our own data using RevMan 5.2, we found the V393A variant to be significantly associated with risk of PD (p = 0.009; Fig 1). This suggests that an association may exist in Asian populations, but larger controlled studies are needed. At the same time, several studies suggest that this association may not exist in Caucasians. The Japanese study failed to detect the V393A variant in Europeans or North Americans [8], and a more recent study also failed to detect the variant in Europeans [18]; another European study detected the variant in only 1 of 1900 patients with PD, but not in any of 788 patients with MSA or 600 healthy controls [19]. Studies are therefore needed to clarify the relevance of this genetic association in Asians and non-Asians.

Subgroup analysis of our data showed significant differences in genotype and minor allele frequencies between EOPD patients and controls, but not between LOPD patients and controls. EOPD usually involves obvious lysosomal and mitochondrial dysfunction, which helps increase oxidative stress; LOPD, in contrast, is usually more associated with synaptic transmission [20]. These findings are consistent with the idea that the V393A variant leads to lower production of coenzyme Q10, resulting in increased oxidative stress. This conclusion should be regarded as preliminary, since it is based on only 167 patients with EOPD in our study. This possibility should be verified in studies with larger numbers of patients with EOPD.

Our study has several limitations. First, although we included only patients with probable PD, diagnosis was not confirmed by pathology in any of our patients. This raises the possibility that some may not have had disease. Second, the relatively small sample of patients with PD meant that the statistical power for calculating genetic associations with PD was only 53.7%. Third, we examined only a limited array of *COQ2* polymorphisms in our patients since we did not sequence the entire gene. We did not detect the M128V or R387X variants, nor did we detect the homozygous CC genotype of the V393A variant. Finally, we did not take into account possible gene-gene or gene-environment interactions that may be confounding our results. These limitations highlight the need for larger studies that consider the complete range of *COQ2* polymorphisms in different ethnicities, especially Asians, as well as possible interactions of the polymorphisms with other genes and the environment.

In conclusion, our study suggests an association between the V393A variant of the *COQ2* gene and risk of PD in Han Chinese and the followed meta-analysis extend this association to the population of east Asia. These results lend genetic support to the hypothesis that oxidative stress underlies the pathogenesis of both neurodegenerative diseases(PD and MSA).
